# Fecal sludge and adjacent drinking water as reservoirs of multidrug resistant ESBL-producing pathogenic *Escherichia coli* in Rohingya Camps, Bangladesh

**DOI:** 10.1038/s41598-026-58907-y

**Published:** 2026-06-22

**Authors:** Mohammed Tanveer Hussain, Amanta Rahman, Md. Sakib Hossain, Mohammad Atique Ul Alam, Nayeema Haque, Faisal Chowdhury Galib, Md. Foysal Abedin, Md. Hajbiur Rahman, Ashrin Haque, M. Moniruzzaman, Mohammad Rafiqul Islam, Mohammed Badrul Amin, Iftekhar Bin Naser, Mahbubul H. Siddiqee, Md. Shafiqul Islam, Zahid Hayat Mahmud

**Affiliations:** 1 Laboratory of Environmental Health, Health Systems and Population Studies Division, International Centre for Diarrhoeal Disease Research, Bangladesh (icddr,b), 68, Shaheed Tajuddin Ahmed Sarani, Mohakhali, Dhaka, 1212 Bangladesh; 2 Department of Pathobiological Sciences, School of Veterinary Medicine, Louisiana State University, Baton Rouge, 70803 USA; 3 Biotechnology Program, Department of Mathematics and Natural Sciences, BRAC University, Mohakhali-66, Dhaka, Bangladesh; 4 Department of Biological Sciences, College of Science, Louisiana State University, Baton Rouge, LA 70803 USA; 5 Department of Microbiology, University of Manitoba, Winnipeg, MB R3T 2N2 Canada; 6 Laboratory of Food Safety and One Health, Nutrition Research Division, International Centre for Diarrhoeal Disease Research, Bangladesh (icddr,b), Dhaka, 1212 Bangladesh; 7 Microbiology Program, Department of Mathematics and Natural Sciences, BRAC University, Mohakhali-66, Dhaka, Bangladesh

**Keywords:** Antibiotic resistance, ESBL, *E. coli*, Fecal sludge, Drinking water, Rohingya camps, Bacteria, Environmental microbiology

## Abstract

Fecal Sludge (FS) is a well-known reservoir for multiple pathogenic organisms and antimicrobial-resistant *E. coli*. Contaminated drinking water (DW) is the primary route through which fecal-oral diseases are spread. This study examined the prevalence of Extended-spectrum beta-lactamase (ESBL)-producing *E. coli* in FS and adjacent DW samples collected within the Rohingya Camps. The DW and treated FS samples were screened for thermotolerant *E. coli* and the isolates were characterized for their ESBL-production ability, presence of major antibiotic resistance (AMR) genes, pathogenic genes, biofilm formation ability and phylogenetic clustering. Among the samples, 88.1% FS and 40.2% DW contained *E. coli*. From the selected isolates, 32.3% of FS and 12.5% of DW were ESBL-positive. Subsequently, 113 ESBL-FS and 56 ESBL-DW isolates were characterized based on their concurrent presence. Upon screening, *bla*_CTX−M_ (81.1%) was the most prevalent AMR gene, followed by *bla*_TEM_ (23.7%) and *bla*_NDM−1_ (14.8%). Enteroaggregative *E. coli* (EAEC) (17.2%) and enterotoxigenic *E. coli* (ETEC) (12.4%) were the most prevalent diarrheagenic pathotypes and 4.7% were extra-intestinal pathogenic *E. coli* (ExPEC) strains. Antimicrobial susceptibility testing (AST) revealed that 98.2% of isolates were multidrug-resistant. At 25 °C, 34.3% of isolates formed strong biofilm. The ERIC (Enterobacterial Repetitive Intergenic Consensus sequences) phylogenetic clustering generated 16 clusters at a 70% similarity index. The detection of pathogenic isolates highlights the risk of severe infections within susceptible populations and a high percentage of multidrug resistance limits the treatment options.

**Supplementary Information:**

The online version contains supplementary material available at https://doi.org/10.1038/s41598-026-58907-y.

## Introduction

Each year diarrheal disease causes the death of around 2 million people worldwide, with more than 500,000 being under the age of 5^1^. Around 88% of diarrheal diseases have been linked to unsafe water supply, hygiene and sanitation, and the children of developing countries have been identified as the primary risk group^2^. *Escherichia coli* is a well-known fecal indicator bacteria and the causative agent of several enteric diseases. The World Health Organization (WHO) Global Burden of Foodborne Diseases estimated > 300 million cases and around 0.2 million deaths attributed to diarrheagenic *E. coli* each year^3^. Although most *E. coli* in nature are harmless and even a part of the natural flora of humans^4^, several strains are known to harbour virulence properties that lead to severe infections. *E. coli* has six well recognized pathotypes that cause diarrhea, namely: ETEC (enterotoxigenic *E. coli*), EHEC (enterohemorrhagic *E. coli*), EAEC (enteroaggregative *E. coli*), EPEC (enteropathogenic *E. coli*), EIEC (enteroinvasive *E. coli*) and DAEC (diffusely adherent *E. coli*). Furthermore, several strains of *E. coli* are known for causing infections outside of the enteric tract and are known as extra-intestinal pathogenic *E. coli* (ExPEC)^5^.

The increased incidence of antimicrobial resistant Enterobacteriaceae, especially *E. coli* has been reported in several studies^6,7^. These infections represent a major public health concern due to their high frequency and their potential to cause severe and sometimes fatal outcomes, especially in children. Extended-spectrum beta-lactamase (ESBL)-producing *E. coli* is a concerning reservoir for potable multidrug resistance (MDR) in bacteria, primarily contributing to severe hospital or community acquired illnesses in countries with inadequate hygiene practices^8^. Discovery of the New Delhi Metallo β-lactamase (NDM)-producing *E. coli* visualizes a scenario where all available antibiotics are rendered ineffective^9^. Furthermore, Cefotaximase-Munich (CTX-M) enzymes which confer resistance to third-generation cephalosporins are a major AMR driver, often located on plasmids that may be transferable to other bacteria^10^. The Temoneira (TEM)-derived ESBLs can hydrolyze extended-spectrum cephalosporins, in addition to narrow spectrum penicillins, restricting the treatment options and use of first-line antibiotics and treatment procedures^11^. The primary mechanism through which resistance is spread in organisms is by horizontal gene transfer (HGT), especially the transfer of plasmids. *E. coli* has been known to acquire genes conferring resistance from organisms in the environment and animals^12,13^.

There are multiple routes through which pathogenic *E. coli* can spread within the population. Primarily, the most frequent route is through the fecal-oral pathway, where infection is caused by the ingestion of contaminated food and water^14^. However, *E. coli* may also spread through person to person contact or through zoonosis, and recent reports have indicated potential airborne transmission^15^. In previous studies, different environmental reservoirs have been shown to harbour high levels of ESBL-producing *E. coli*, including drinking water (DW), wastewater and recreational water^16–18^. The contamination of household DW is one of the primary ways in which individuals ingest ESBL-producing *E. coli*, and it has been linked to an increased incidence of diarrheal diseases^16,19^. Till now, few studies have targeted ESBL-producing *E. coli* in household DW^20–22^. Furthermore, fecal sludge (FS) is a well-known repository of *E.coli*, and pathogens harbouring high levels of AMR genes have been isolated from human fecal material^23^. A cause for concern is the inefficiency of the FS treatment plants in removing infectious pathogens, which can be transmitted into the soil and the food chain through the use of wastewater and FS for agriculture^24–26^. A few studies have isolated ESBL-producing *E. coli* from FS and related human waste matrices, highlighting fecal sludge as an important environmental reservoir of antimicrobial-resistant bacteria; however, the number of studies specifically investigating these organisms in fecal sludge remains limited^17,27–29^.

In Bangladesh, diarrheal diseases are endemic and remain a major public health concern; in 2020 alone, more than 36,000 people died of diarrheal diseases in Bangladesh^30^. The leading pathogen contributing to the most diarrheal cases was *E. coli*^31^, which is a reservoir of β-lactam resistance and is responsible for its spreading to surrounding microbes^32^. As a result, it is important to detect and characterize ESBL-producing *E. coli* from DW among densely populated community settings or areas with a lack of potable DW and poor maintenance of hygienic conditions^33,34^. Consequently, monitoring ESBL-producing *E. coli* in FS may help in controlling the outbreak due to ESBL-producing *E. coli*. Although previously conducted studies have isolated ESBL-producing *E. coli* from FS and DW of the Rohingya camps in Cox’s Bazar^16,17^, there remains an urgent need for effective re-evaluation of contamination levels. Furthermore, there has been no effective comparison between the reservoirs present in the same geographical location, and often within proximity to one another. The comparison may help identify pathways of cross contamination, given that improperly treated FS can leach into water sources through seepage and runoff. It may also help design targeted interventions such as fecal management to prevent improper disposal and enhanced water purification systems to remove resistant bacteria. The public health implications of these findings may help communicate the urgency of water safety measures in such settings and highlight the role of environmental reservoirs as a breeding ground for these pathogens. Hence, we targeted isolating ESBL-producing *E. coli* from household DW and FS in Rohingya camps in Cox’s Bazar. The isolates were characterized for their ESBL activity, antimicrobial resistance, and presence of major AMR genes, alongside their biofilm formation capacity.

## Methodology

### Sampling locations and sample collection

We collected FS and DW samples for seven months from September 2022 to March 2023. The study was conducted in Rohingya Camps, Cox’s Bazar with prior approval of the Refugee Relief and Repatriation Commissioner (RRRC). The Rohingya refugee camps in Cox’s Bazar were chosen due to their high population density, reliance on on-site sanitation systems, and close proximity between fecal sludge management infrastructure and drinking water sources, which together create a high risk for fecal contamination and antimicrobial resistance transmission. In total, 63 fecal sludge treatment plants (FSTPs) were selected for FS collection, and corresponding households were selected for DW collection; all selected households were within 100 m in proximity to each FSTP. Altogether, 126 FS (from 63 FSTPs) samples were collected from both the inlet and outlet, alongside 189 corresponding DW samples from nearby households. In this study, drinking water samples were collected as point-of-use household drinking water, which is primarily sourced from groundwater and stored in household containers prior to consumption. For both FS and DW, sterile wide mouth PPCO bottles with proper label (NALGENE, NY, USA) were used and 500 ml of samples were collected. Following collection, samples were transported to the Laboratory of Environmental Health, icddr, b, Dhaka from Cox’s Bazar, keeping a temperature range of 4℃-10℃^17^.

### Sample processing

The sample processing was conducted within 24 h of sample collection and samples were maintained at 4℃ prior to processing. During processing, the FS samples were serially diluted upto 10^− 6^ using sterile 0.85% NaCl (normal saline). A 100 mL volume of each dilution was filtered using a 0.22 μm nitrocellulose membrane (Sartorius, Goettingen, Germany) using a Millipore filter unit (Millipore, Darmstadt, Germany). For DW samples, 100 mL of the samples was filtered without any prior dilution. Filters were then placed onto the modified Thermotolerant *E. coli* (mTEC) agar (BD Difco, USA). Initial incubation was carried out at 35$\:\pm\:$0.5°C for 2$\:\pm\:$0.5 hrs with second round of incubation for 22$\:\pm\:$2 hrs at 44.5$\:\pm\:$ 0.2°C^28,35^.

### ESBL-positive *E. coli* identification and isolation

Following incubation, redish or magenta colonies on mTEC agar were indicative of thermotolerant *E. coli*. For adequate representation, ≤ 20 colonies from each contaminated DW and FS sample were randomly selected and inoculated into CHROMagar™ ESBL (CHROMagar, Paris, France) based on distinct colony morphology and color on the mTEC agar plates. A total of 1588 isolated colonies (1046 from FS and 542 from DW) were inoculated onto CHROMagar™ ESBL (CHROMagar, Paris, France) using the patch inoculation method, followed by incubation at 35$\:\pm\:$0.5°C for 18–24 h^16^. The growth on the media is indicative of ESBL production, whereas dark pink to reddish colonies are indicative of *E. coli*. For further analysis, DW samples with contamination and their corresponding FS samples were chosen. Following confirmation, the isolates were streaked onto MacConkey agar (BD Difco, NJ, USA) to obtain single colonies and enriched overnight using Luria Bertani (LB) broth and stored using a 30% glycerol stock preparation.

### PCR-based *bla* gene detection

The boiling lysis method was used for DNA extraction from the isolates following a protocol published previously^36^. Presence of *bla* genes, namely *bla*_CTX−M_, *bla*_TEM_, *bla*_SHV_, and *bla*_OXA_, was identified using the extracted DNA following a protocol described previously^37^. For the isolates containing *bla*_CTX−M_, the isolates were tested to differentiate between the presence of different CTX-M groups, namely *bla*_CTX−M−1_ group, *bla*_CTX−M−*9*_ group, *bla*_CTX−M−2_ group, and *bla*_CTX−M−8_ group using protocols published previously^16^. Further, all the isolates were tested for the presence of *bla*_*NDM−1*_ using methods described previously^38^. The reaction mixtures were amplified using a Bio-Rad T100™ Thermal Cycler (Bio-Rad, CA, USA) and resolved using agarose gel electrophoresis utilizing a 1% agarose gel. Primer sequences and their product sizes are listed in Supplementary Table S1.

### Diarrheagenic *E. coli* detection

The isolates were assessed for the presence diarrheagenic-associated genes. The presence of genes encoding certain diarrheagenic pathotypes was tested including *aat* (anti-aggregation protein transporter) and *aaiC* (aggR-activated island) for EAEC, *eae* (attaching and effacing) and *bfp* (bundle forming pilus) for EPEC, *lt* (heat-labile) and *st* (heat-stable) for ETEC, *ipaH* (invasion plasmid antigen H) and *ial* (invasion-associated locus) for EIEC^39,40^. The PCR was carried out as a multiplex for the four pathotypes using a protocol published previously^17^. Further, all the isolates were tested for genes coding for *stx1* and *stx2* (Shiga toxin) for EHEC using a previously published protocol^41,42^.

### Detection of ExPEC associated virulence genes

The ESBL-producing isolates were investigated for seven ExPEC-related genes, namely, *iutA* (iron acquisition system), *focG* (F1C fimbriae), *hlyD* (cytolytic protein toxin), *kpsMII* (group 2 polysaccharide capsule), *sfaS* (S fimbriae), *papA* (P fimbriae) and *afa* (afimbrial adhesins). The genes were detected via two multiplex PCRs using previously published protocols^43^. If an isolate was found to harbor three or more of the genes listed above, the isolate was termed as an ExPEC strain as per pre-determined criteria^17,43,44^.

### Determination of antibiotic susceptibility profiles

Antimicrobial sensitivity profiles of the isolates were assessed as per the recommendation of Clinical Laboratory Standards Institute (CLSI) guidelines^45^. Resistance profiles were screened against 20 different antibiotics belonging to 19 classes (Supplementary Table S2), using commercial disks (Thermo Fisher Scientific™, USA). The concentrations of antibiotics used can be found in Supplementary Table S2. The assay was replicated three times using *E. coli* ATCC 25922 as quality control.

### Investigating molecular relatedness among ESBL-producing isolates

To investigate the genetic relatedness, ERIC (Enterobacterial Repetitive Intergenic Consensus sequences) PCR was performed using the primer listed in Supplementary Table S1, using the protocols published previously^46^. PCR amplification was separated using a 2% agarose gel, with Invitrogen 1 kb plus ladder (Thermo Fisher Scientific™, Waltham, USA). Gel image analysis was done using GelJ v.2.0^47^, and for image normalization, the Gaussian regression method was utilized. The patterns resulting from the ERIC-PCR were clustered to generate a phylogenetic tree using the dice coefficient and the unweighted pair group method using arithmetic averages (UPGMA) with a 1.0% tolerance value. The genetic distances are computed by the UPGMA algorithm that builds a distance-based hierarchical tree.

### Biofilm formation detection

Biofilm forming capacity of the isolates was tested using the quantitative adherence assay^48^. The isolates were cultured overnight in Luria-Bertani (LB) broth at 37 °C. Following enumeration, 2 µL of the enriched culture was pipetted onto a sterile 96-well microtiter plate (Corning Life Sciences, NY, USA) containing 198 µL of sterile LB. For the negative control, uninoculated LB broth was used. The plates were then incubated at two tested temperatures of 25 °C and 37 °C for a period of 48 h. Following incubation, the plates were rinsed thrice with 200 µL PBS, and dried at room temperature in an inverted position. The plates were then dyed using 50 µL of 0.1% crystal violet for 15 min. After staining, the plates were then rinsed again thrice with PBS. After drying, the captured crystal violet in biofilms was solubilized with 200 µL of 5% isopropanol. The plates were then read at a wavelength of 590 nm with a microtiter plate reader (BioTek, Vermont, USA), to determine their optical density (OD). The experiment was repeated thrice and the average OD was used to categorize the isolates as either strong, weak, moderate or non-biofilm former as per previously published protocol^49^. Previous studies have successfully used LB broth for culturing *E. coli* as the growth medium prior to the biofilm assay and assessing biofilm formation^50–53^; hence we followed a validated protocol using LB medium for reproducible results.

### Statistical analysis

For statistical assessment, data were converted to binary values (1 for presence, 0 for absence). The R programming language (Version 4.1.1) was used for analysis^54^. The ‘cor’ function calculated correlations, and Pearson correlation was utilized in ‘corr.test’ to assess their significance (p < 0.05). Significant correlations were visualized with the ‘corrplot’ function^55^. Hierarchical clustering was conducted with the ‘pheatmap’ function using Ward’s method for distance matrices^56^. Genes with 100% resistance to ampicillin, cefotaxime, and cefuroxime were excluded from the correlation plot due to a lack of significance.

## Results

### High prevalence of ESBL-producing *E. coli* in fecal sludge samples and adjacent drinking water

In total, 126 FS (inlet and outlet) samples were collected, alongside 189 corresponding DW samples from households within proximity to the treatment plants. From these, 111/126 (88.1%) FS and 76/189 (40.2%) DW samples were contaminated with *E. coli*. For adequate representation, ≤ 20 *E. coli* isolates from each contaminated sample were randomly selected and inoculated onto CHROMagar™ ESBL and positive colonies were picked for further analysis. A total of 338/1046 (32.3%) isolates from FS and 68/542 (12.5%) from DW were positive for ESBL production. For subsequent molecular and phenotypic analyses, ESBL-positive isolates were selected from drinking water samples and their corresponding fecal sludge samples obtained from the same sampling sites to allow paired source comparison. Among them, 113 *E. coli* isolates were selected from FS, and 56 isolates were selected from DW. The presence of ESBL-producing *E. coli* contamination was rather diverse, with 85/126 (67.5%) FS and 27/189 (14.3%) DW samples being contaminated. For the inlet and outlet comparisons, a total of 61/63 (96.8%) inlets were contaminated with *E. coli* and 47/63 (74.6%) were positive for ESBL-producing *E. coli*. On the other hand, 50/63 (79.4%) outlet samples contained *E. coli* with 38/63 (60.3%) positive for ESBL-producing *E. coli*. A two-proportion test indicated that this reduction was not statistically significant (*p* = 0.08). The count of *E. coli* isolates is represented in Supplementary Table S3.

### *bla*_CTX-M_ was more prevalent in both drinking water and fecal sludge isolates

**Fig. 1:**
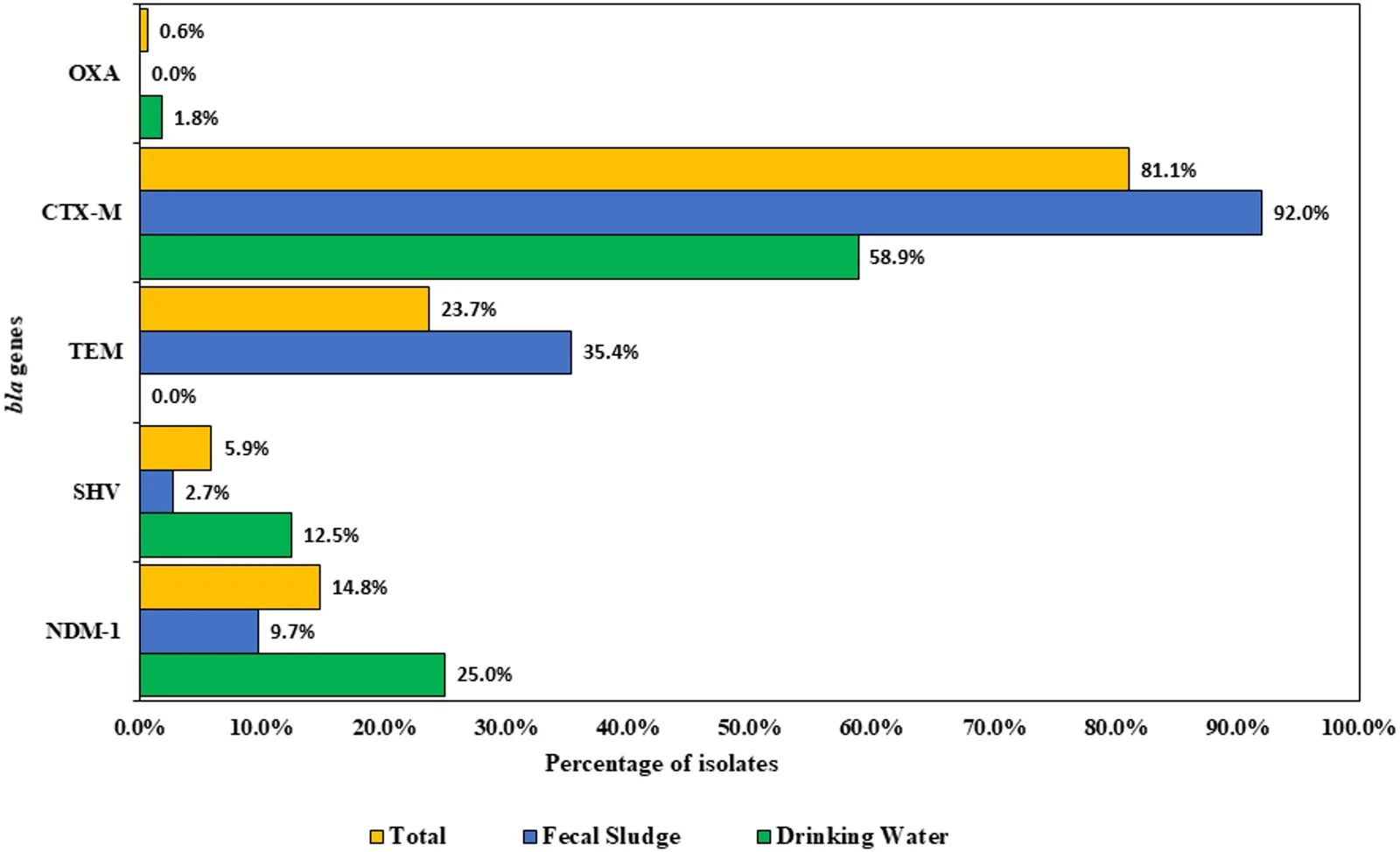
Distribution of major antibiotic resistance genes within the EBSL *E. coli*.

The ESBL-producing *E. coli* were analyzed for β-lactamase and ESBL genes using PCR. Among them, *bla*_CTX−M_ was present in 137/169 (81.1%), *bla*_TEM_ in 40/169 (23.7%), *bla*_SHV_ in 10/169 (5.9%) and *bla*_OXA_ in 1/169 (0.6%) of the isolates. Furthermore, 25/169 (14.8%) isolates were positive for *bla*_NDM−1_ (Fig. 1). CTX-M cluster breakdown shows the most prevalent was the *bla*_CTX−M−1_ group, found in 135/169 (79.9%) isolates, followed by *bla*_CTX−M−9_ group in 9/169 (5.3%) isolates. On the other hand, the *bla*_CTX−M−2_ or *bla*_CTX−M−8_ group was not present in the ESBL-producing *E. coli* isolates.

The DW isolates were found to harbour a higher prevalence of *bla*_CTX−M_ (59%), *bla*_NDM−1_ (25%), *bla*_SHV_ (12.5%) and *bla*_OXA_ (1.8%). On the other hand, in FS isolates, *bla*_TEM_ (35.4%) and *bla*_CTX−M_ (92%) were more prevalent (Fig. 1). Find representative gel images in the ‘Supplementary File’ Figs. S1 and S4.

### Prevalence of diarrheagenic and extra-intestinal pathogenicity associated genes in fecal sludge and drinking water isolates

**Fig. 2:**
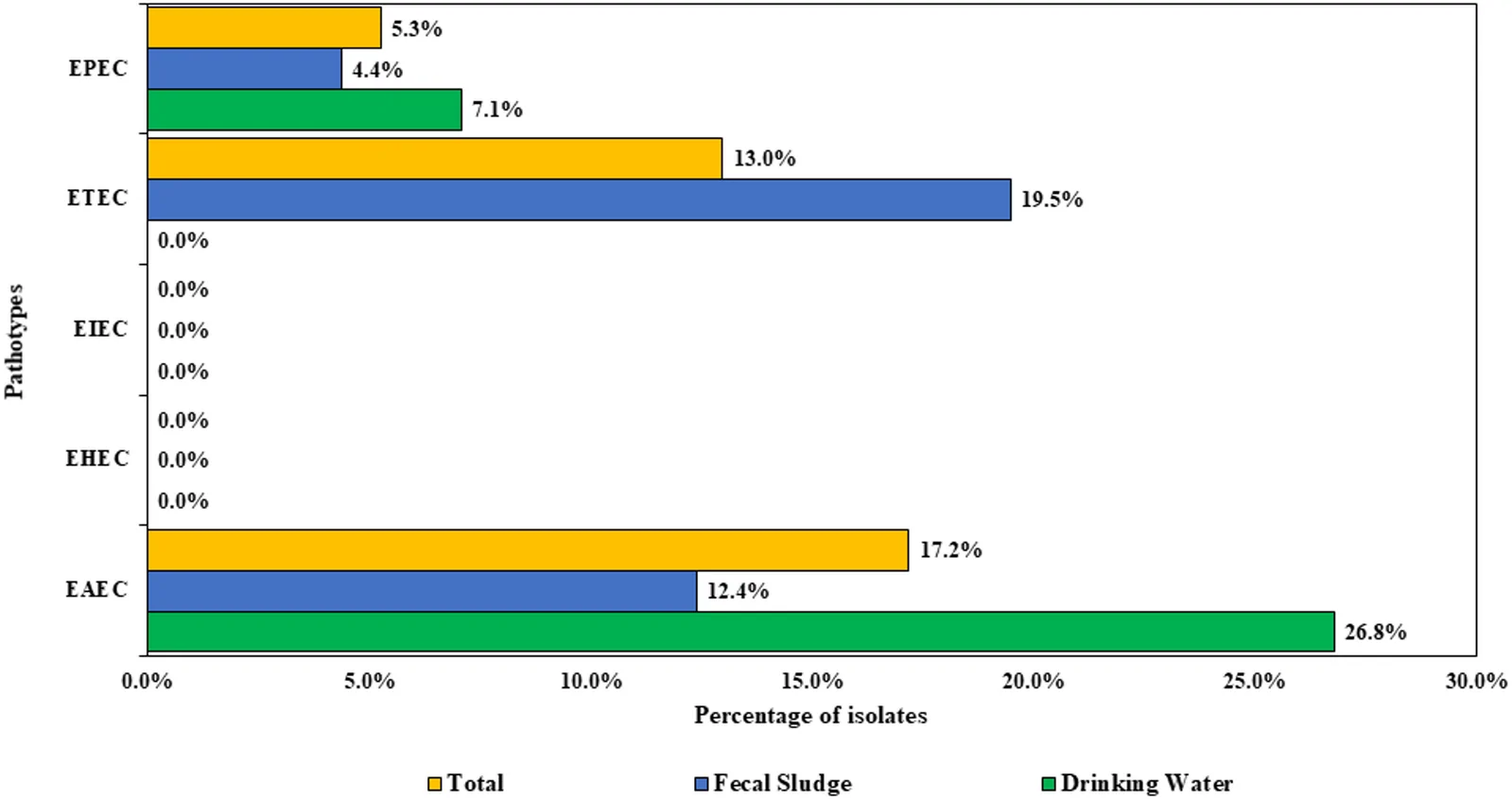
Prevalence of diarrheagenic pathotypes among the ESBL-producing *E. coli* isolates.

Among diarrheagenic genes, a minimum of one virulence gene was found in 60/169 (35.5%) of the isolates. The gene *aaiC* was majorly prevalent being detected in 29/169 (17.2%) of the isolates, followed by *estA* in 21/169 (12.4%), *eltB* in 15/169 (8.9%), *bfpA* in 8/169 (4.7%) and *eae* in 1/169 (0.6%) isolates. However, none of the isolates were positive for the *aat*, *iaa*, *ipaH*, *stx 1* and *stx 2* genes. Among the pathotypes detected, EAEC was 29/169 (17.2%), ETEC 22/169 (13%) and EPEC 9/169 (5.4%) (Fig. 2). With respect to sample types, genes related to EAEC (26.8%) and EPEC (7.1%) were more prevalent in DW isolates, whereas genes related to ETEC (19.5%) were found in higher percentages among FS isolates (Fig. 2).

**Fig. 3:**
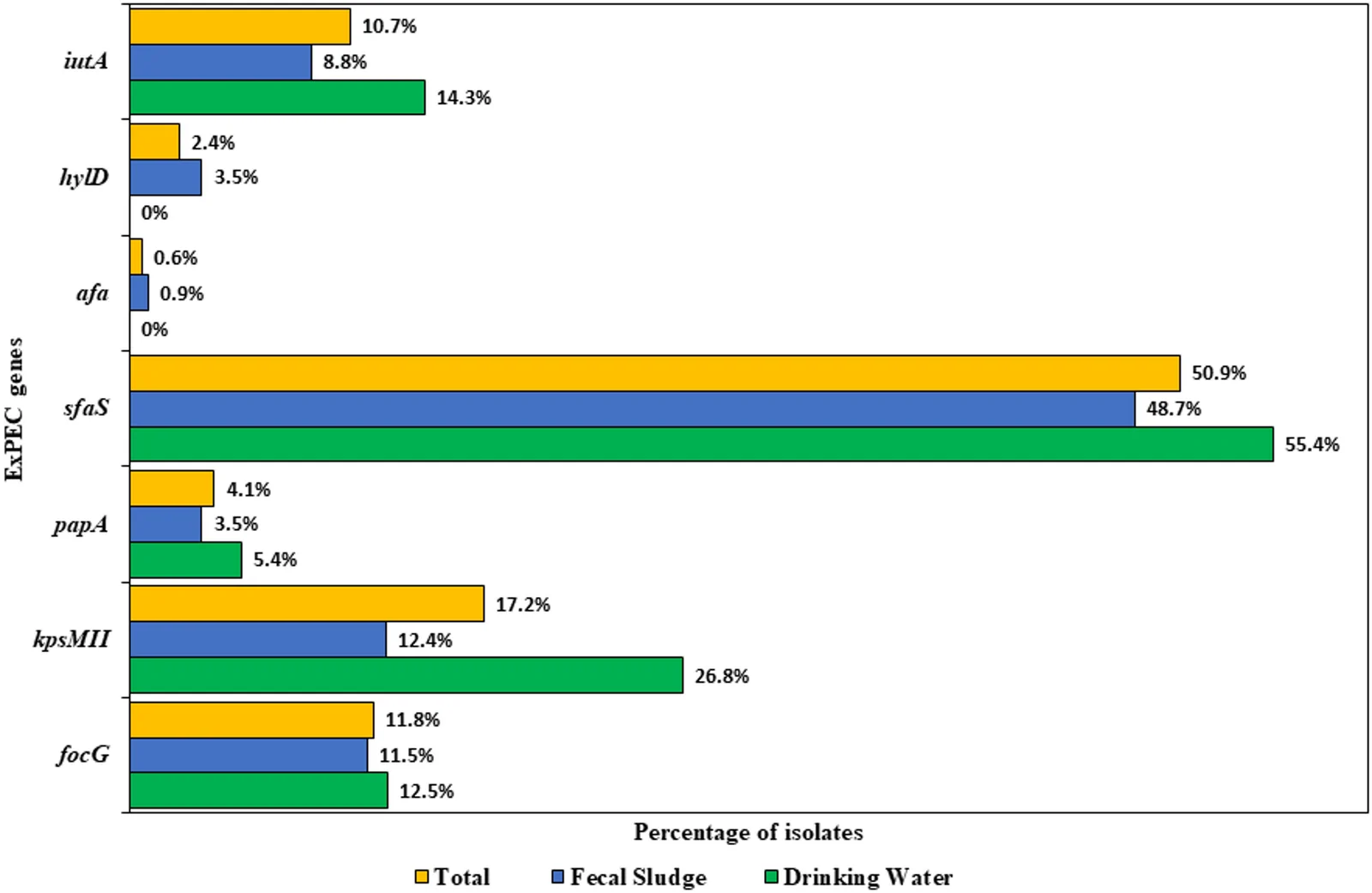
Prevalence of ExPEC genes among the ESBL-producing *E. coli* isolates.

In the case of extra-intestinal pathogenicity, all seven virulence factors were detected among the tested isolates. For the virulence determinants, *sfaS* was detected in 86/169 (50.8%) isolates, *kpsMII* in 29/169 (17.2%), *focG* in 20/169 (11.8%), *iutA* in 18/169 (10.6%), *papA* in 7/169 (4.2%), *hlyD* in 4/169 (2.4%) and *afa* in 1/169 (0.6%) isolates (Fig. 3). In terms of coexistence, the DW isolates were found to harbour a higher prevalence of *focG* (12.5%), *kpsMII* (26.8%), *papA* (5.4%), *sfaS* (55.4%) and *iutA* (14.3%). On the contrary, the FS isolates had a higher prevalence of the *afa* (0.9%) and the *hlyD* (3.5%) genes (Fig. 3). Although 60/169 (35.5%) isolates carried at least one ExPEC gene, 8/169 (4.7%) isolates were classified as ExPEC strains harbouring at least three genes^17,43,44^. Find representative gel images in ‘Supplementary File’ Figs. S2 and S3.

### Prevalence of multidrug resistance among the ESBL-producing *E. coli*

**Fig. 4:**
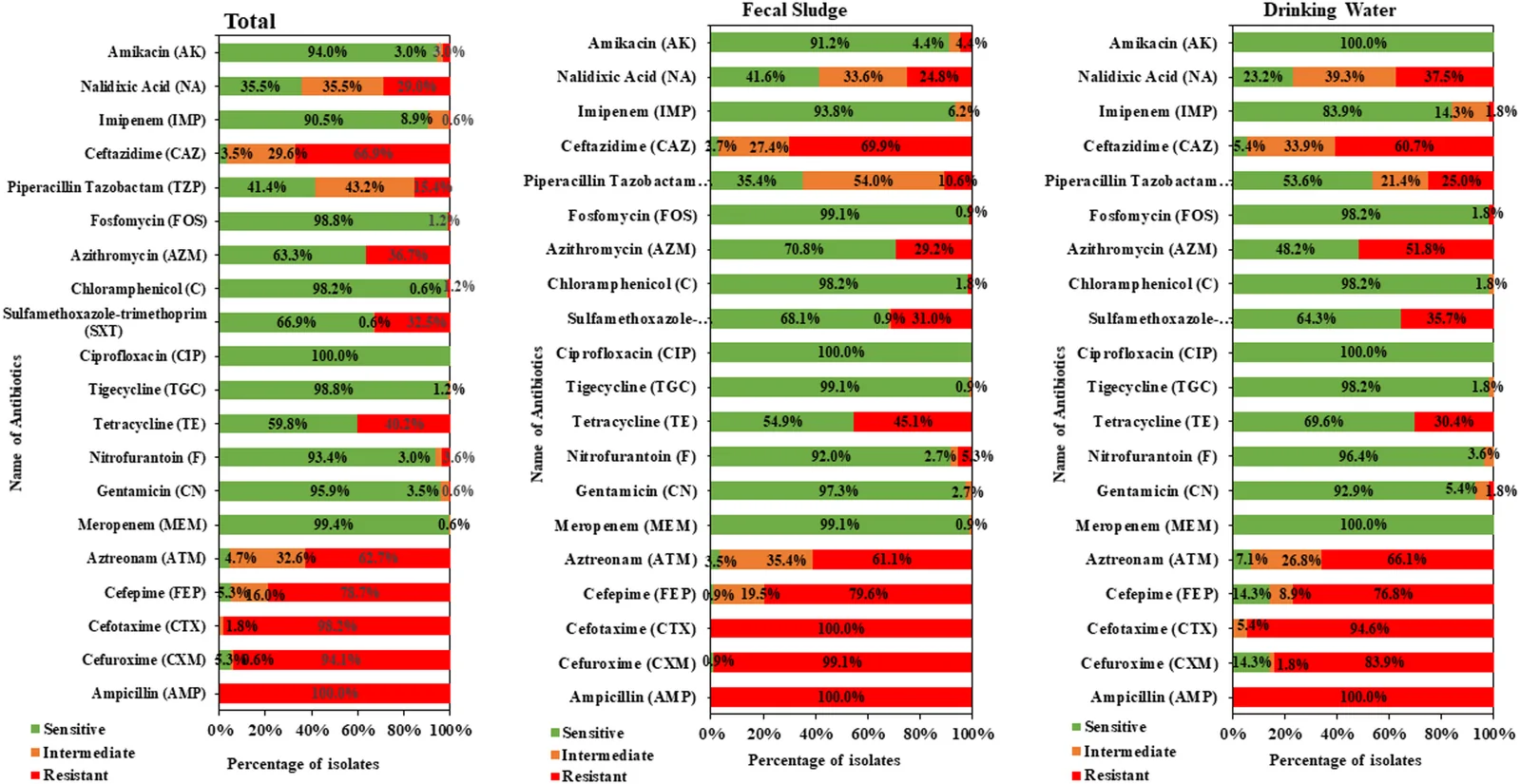
Antibiotic susceptibility profiles of the ESBL-producing *E. coli* isolates against 20 commercially used antibiotics. (**A**) shows resistance status of the entire population of isolates, (**B**) signifies antibiotic resistance among the FS isolates and (**C**) signifies antibiotic resistance among the DW isolates.

Drug resistance profiles were examined using the CLSI recommended method. Notably, 169/169 (100%) of isolates were resistant to ampicillin, 166/169 (98.2%) to cefotaxime, 159/169 (94.1%) to cefuroxime, whereas the rest of the isolates showed diverse resistance patterns to additional antibiotics evaluated (Fig. 4). The most interesting finding was that a total of 166/169 (98.2%) isolates were found to be MDR with resistance to ≥ 3 drug classes. In comparison to DW isolates, the FS isolates had a greater proportion of resistance to cefuroxime, cefotaxime, cefepime, nitrofurantoin, tetracycline, chloramphenicol, ceftazidime and amikacin (Fig. 4). On the other hand, the DW isolates demonstrated a higher prevalence of resistance to aztreonam, gentamicin, sulfamethoxazole-trimethoprim, azithromycin, fosfomycin, piperacillin-tazobactam, imipenem and nalidixic acid (Fig. 4).

### Biofilm forming capability of ESBL-producing *E. coli*

**Fig. 5:**
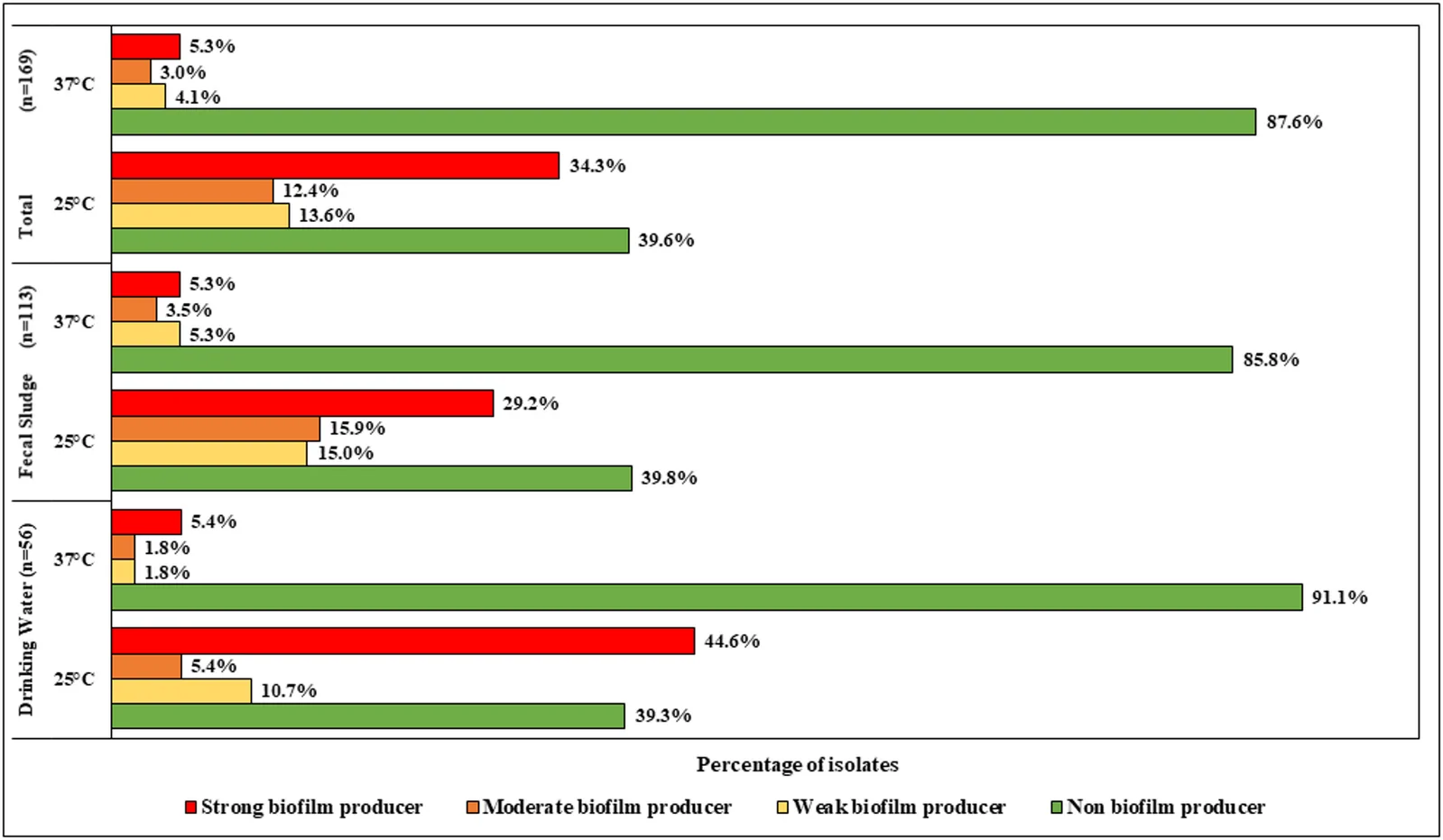
The biofilm formation capacity of the ESBL-producing *E. coli* isolates, the isolates have been divided according to temperature as well as isolation source.

The degree of biofilm formation by the isolates was observed at temperatures of 25 °C and 37 °C. A greater number of isolates showed higher degrees of biofilm formation at 25 °C in comparison to 37 °C. The percentages of isolates generating various degrees of biofilm are represented in Fig. 5. At a temperature of 25 °C, 58/169 (34.3%) were strong, 21/169 (21%) were moderate, 23/169 (13.6%) were weak and 67/169 (39.6%) were non-biofilm forming isolates. In the case of 37 °C, 9/169 (5.3%) isolates exhibited strong, 5/169 (3%) moderate, 7/169 (4.1%) weak and 148/169 (87.6%) non-biofilm formation. It was observed that DW isolates formed higher degrees of biofilm at 25 °C and 37 °C, in comparison to FS isolates (Fig. 5).

### Phylogenetic clustering reveals similarities between drinking water and fecal sludge isolates

**Fig. 6:**
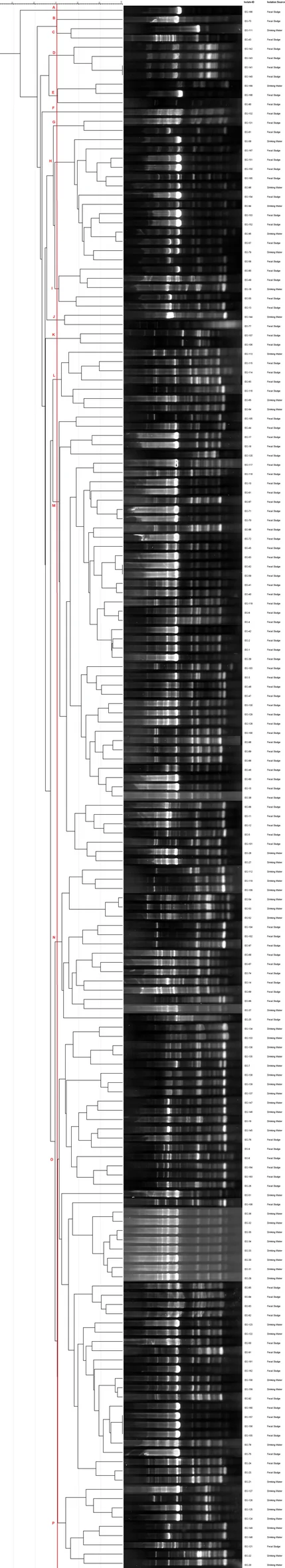
Dendrogram generated based on the ERIC profiles of the isolates using GelJ V2.0 software, the letters indicate individual clusters computed at a 70% similarity index.

At a 70% similarity index, the ERIC profiles of the isolates resulted in a total of 16 clusters. The isolates generated 4–14 amplicons, with 77 and 11 isolates positive for 700 bp and 2000 bp amplicons respectively. The dendrogram revealed multiple isolates from fecal sludge and drinking water clustered together, indicating genetic similarity between FS- and DW-derived isolates from surrounding areas. The maximum number of isolates (50) was found in cluster ‘O’ with 17 isolates obtained from DW and 33 isolates obtained from FS (Fig. 6). The clusters ‘A’, ‘B’ and ‘F’ contained one isolate each obtained from FS. The isolates EC-158, EC-159 and EC-92 were grouped under cluster ‘O’ and exhibited a high percentage of similarity. Likewise, EC-165 and EC-96, EC-95 and EC-57 grouped under cluster ‘H’ also exhibited high amounts of sequence similarities (Fig. 6). Find the representative gel image in ‘Supplementary File’ Fig. S5.

### Hierarchical clustering reveals relatedness between drinking water and fecal sludge isolates

**Fig. 7:**
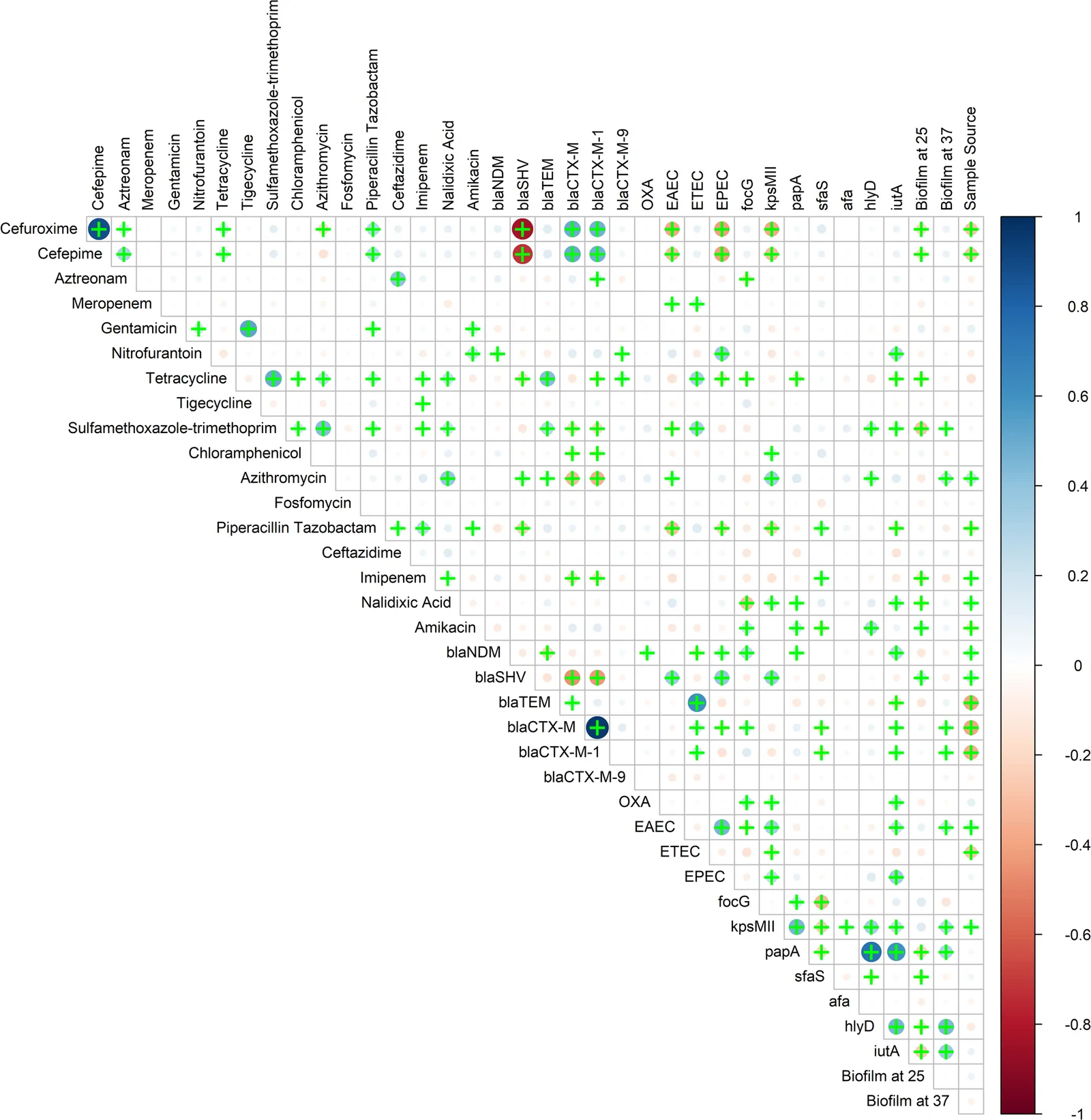
The correlation matrix illustrates relationships between phenotypic features (antimicrobial resistance and biofilm formation) as well as genotypic characteristics (antimicrobial resistance genes and pathogenic genes). Areas marked by white spaces indicate a lack of correlation. Blue circles denote positive correlations, while red circles represent negative correlations. The size and intensity of the colors reflect the magnitude of the correlation coefficient. Statistically significant associations (*p* < 0.05) are marked with a “green plus” symbol, as determined by the Chi-square test.

The relationships between sampling source, genotypic and phenotypic traits were investigated using hierarchical clustering (along with heatmap) and correlation matrix analysis. The analysis revealed positive associations between beta-lactamase gene presence and phenotypic resistance (Fig. 7, *p* < 0.05). The resistance to tetracycline and sulfamethoxazole-trimethoprim was positively correlated to the *bla*_TEM_ gene (Fig. 7, *p* < 0.05). Furthermore, analysis of the resistance patterns revealed associations and the co-occurrence of resistance. For instance, the resistance to piperacillin-tazobactam had a positive correlation with cefuroxime, cefepime, gentamicin, tetracycline, tigecycline, sulfamethoxazole-trimethoprim and chloramphenicol. Likewise, the resistance to cefepime was correlated with the resistance to cefuroxime, aztreonam with cefuroxime and cefepime, tigecycline with gentamicin, sulfamethoxazole-trimethoprim with tetracycline and fosfomycin, ceftazidime with aztreonam, and nalidixic acid with azithromycin. Additionally, the presence of virulence factors was positively linked to the existence of resistance to several antibiotics. Among them, the presence of the ETEC phenotype was positively correlated with resistance to tetracycline and sulfamethoxazole-trimethoprim, whereas the EPEC phenotype was associated positively with resistance to nitrofurantoin. Furthermore, the presence of phenotypic resistance was positively correlated with the presence of resistance genes, for example, the presence of *bla*_CTX−M_ and *bla*_CTX−M−1_ was positively correlated to cefuroxime and cefepime resistance. Besides, positive correlation was found between *bla*_TEM_ with resistance against tetracycline and sulfamethoxazole-trimethoprim. Additionally, the ability to form biofilm was positively correlated with virulence factors. Biofilm formation at 37 °C showed a favorable correlation with the existence of both *iutA* and *hlyD* genes (Fig. 7, *p* < 0.05). The correlation coefficient among the variables is shown in Supplementary Table S4.

**Fig. 8:**
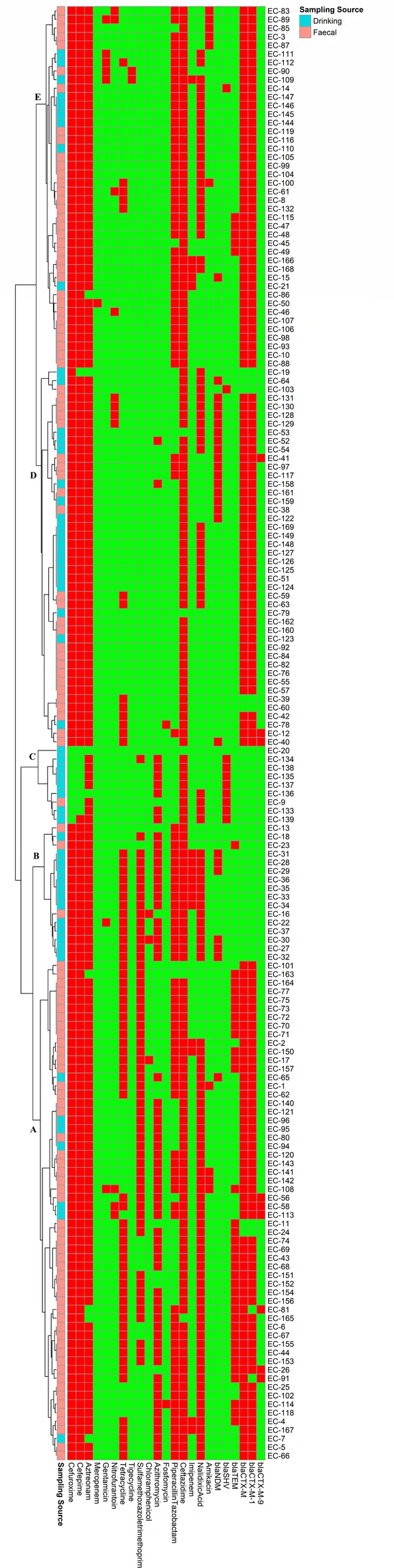
The heatmap and hierarchical clustering of *E. coli* isolates are organized based on their phenotypic traits (antibiotic resistance) and genotypic profiles (antibiotic resistance genes), highlighting the variability among isolates. The color red indicates the presence of resistance or genes, while green signifies their absence. Within the heatmap, the left side consists of a color key that distinguishes between different sources, with DW represented in bright cyan and FS samples in light coral, along with corresponding sampling points. Hierarchical clustering was conducted using Wald’s method alongside a binary distance matrix. The clusters are labeled A through E, representing the five primary clusters discussed in the text.

The ESBL-producing isolates were clustered and segregated according to their phenotypic and genotypic antimicrobial resistance profiles, using hierarchical clustering. In total, five key clusters were recognized. Cluster-A was the largest and contained 58 isolates, with 7 DW and 51 fecal isolates. Cluster B consisted of 16 isolates, with 3 isolates from FS and 13 from DW. Cluster- C was formed with the least number of isolates, with 8 DW and 1 FS isolate. Most notably, Cluster-D consisted of 19 DW and 25 FS isolates (Fig. 8). Lastly, Cluster-E consisted of 33 FS isolates and 9 DW isolates. Interestingly, isolates from different FS treatment plants and DW were clustered together, exhibiting their similarities even when isolated from different geographical locations. For example, isolates EC-52, EC-53 were clustered with EC-128, 129, although they were isolated from different DW sources that were in proximity to a different plant. Interestingly, the isolates EC-56 and EC-58 were clustered together, with EC-56 being isolated from FS and EC-58 being isolated from proximal DW, suggesting that the contamination in DW/FS may be from the same origin (Fig. 8).

## Discussion

The recent rise in antimicrobial resistance has led the World Health Organization (WHO) to place ESBL-producing *E. coli* on the pathogen priority list^57^. Fecal contamination of surface water, wetlands, and potable water plays a critical role in the environmental dissemination of antibiotic-resistant bacteria, as human and animal feces can introduce both resistant organisms and resistance genes into aquatic environments^58^. These contaminated water bodies may act as reservoirs that facilitate the persistence, amplification, and spread of AMR through human exposure and environmental transmission pathways. Fecal sludge, in particular, can harbor pathogenic bacteria at concentrations substantially higher than other environmental reservoirs, posing a significant public health risk^59^. Furthermore, ESBL-producing *E. coli* in DW is especially concerning, as it represents a direct route for fecal–oral transmission of resistant infections^16^. It remains important to analyze ESBL-producing *E. coli* from DW in community settings, where these organisms pose elevated risks of waterborne diseases because of the prevalent unhygienic living conditions. Therefore, in this study, we aimed to isolate and characterize ESBL-producing *E. coli* from FS samples and proximal DW in Rohingya camps, Bangladesh to identify the similarities and differences between these isolates in terms of gene presence, biofilm formation ability and antimicrobial susceptibility.

In this study, 47/63 (74.6%) of the FS inlet samples and 38/63 (60.3%) of the outlet samples contained ESBL-producing *E. coli*, with the difference indicating a p-value of 0.08 at a 5% level of significance. This suggests that while the fecal sludge treatment process may contribute to a partial reduction of ESBL-producing *E. coli*, the treatment may not be sufficiently effective in consistently eliminating these resistant bacteria. The persistence of ESBL-producing *E. coli* in outlet sludge highlights the potential risk of environmental dissemination if treated sludge is released or reused without adequate safeguards. Compared with a previous study conducted in Rohingya camps of Cox’s Bazar, which reported ESBL-producing *E. coli* in 78% (42/54) of inlet samples and 69% (37/54) of outlet samples, the present findings suggest a slight overall decrease in the prevalence of ESBL-producing *E. coli*^17^. However, the difference is less than 10% and the overall decrease may be attributable to a larger sample size used in this study. The continuous presence of ESBL-producing *E. coli* within these FS treatment plants acts as a persistent reservoir of these organisms and may be the underlying reason for waterborne diseases within the area^60^. This study examined the surrounding point-of-use DW close to these FS treatment plants and found 76/189 (40.2%) of the samples were contaminated with *E. coli.* When compared to previous studies within the region, the results suggest an overall increase in prevalence of point-of-use DW^16^. With regards to ESBL production, 27/189 (14.3%) of these samples contained ESBL-producing *E. coli*¸ and the results are comparable to those detected previously^16^. Previous studies have reported considerable variation in the prevalence of ESBL-producing *E. coli* in drinking water across different regions of the world. Higher prevalence rates are often reported in low- and middle-income countries where sanitation infrastructure, wastewater management, and drinking water protection measures may be limited, with higher rates detected in African countries and lower rates observed within high-income countries such as the USA, with strict water quality regulations and monitoring systems^61–63^. The difference in rates observed may be attributed to critical factors such as economic status, sanitation policies and strict mandates on water quality guidelines and highlights the importance of improving water safety and sanitation infrastructure in overcrowded settings such as Bangladesh. To reduce *E. coli* contamination in drinking water at the household level, point-of-use water treatment methods such as chlorination, boiling, or filtration can effectively eliminate microbial contaminants. Ensuring safe water storage, using narrow-neck containers with lids and taps, can prevent recontamination after collection.

Among the 169 ESBL-producing *E. coli* isolates, multiple β-lactamase genes were detected. Among the resistance genes, *bla*_CTX−M_ was the most prevalent, being detected in 137/169 (81.1%) isolates followed by *bla*_TEM_ in 40/169 (23.7%), *bla*_SHV_ in 10/169 (5.9%) and *bla*_OXA_ in 1/169 (0.6%) of the isolates. Previous studies have shown that the *bla*_CTX−M_ type genes are the most common within the ESBL group, overtaking *bla*_TEM_ and *bla*_SHV_ since the early 2000s^64^. Furthermore, studies have also shown that *bla*_CTX−M_ is the most common ESBL gene detected within the ESBL-producing isolates^16,17^. In comparison to previous studies conducted in the same geographical setting between 2019 and 2021, the rates of *bla*_TEM_ and *bla*_OXA_ suggested an overall decrease, whereas increased rates were observed for *bla*_SHV_^16,17^. These differences may reflect temporal changes in antimicrobial selection pressure, shifts in antibiotic usage patterns within the camps, which have been reported in refugee and humanitarian settings where access to antibiotics and prescribing practices may vary over time. Variations in sanitation infrastructure, fecal sludge management practices, and environmental exposure pathways within the Rohingya camps may also influence the distribution and persistence of ESBL genes in environmental reservoirs. Previous studies have highlighted that overcrowding, limited sanitation facilities, and evolving water, sanitation, and hygiene (WASH) interventions in refugee settings can contribute to the environmental dissemination and persistence of antimicrobial-resistant bacteria^65–69^. WASH includes access to safe drinking water, improved sanitation facilities, and hygiene practices such as handwashing. These elements play a vital role in reducing fecal contamination and preventing the spread of enteric pathogens. The *bla*_NDM−1_ gene was detected in 25/169 (14.8%) isolates and this gene, along with its variants, is regarded as a major health concern, given its ability to inactivate routinely employed and widely available β-lactam antibiotics^70^. This study found the presence of multiple *bla*_CTX−M_ types, with *bla*_CTX−M−1_ group being the most prevalent. Previous studies have shown the *bla*_CTX−M−1_ group to be the most common within ESBL isolates, and the prevalence observed in the present study is similar to those detected previously^17,71^. Furthermore, our study indicated the presence of *bla*_CTX−M−9_ group within the ESBL isolates, which has previously been undetected within the FS and DW isolates^16,17^. These findings highlight the dynamic nature of ESBL gene profiles in humanitarian settings and underscore the importance of continued environmental surveillance to track emerging resistance patterns and inform targeted WASH and antimicrobial stewardship interventions.

The study of virulence factors helps identify the prevalence of pathogenic isolates within these bacterial reservoirs, and where gastrointestinal infections are prevalent, pathogenic *E. coli* may significantly contribute to the mortality rates. In this study, a minimum of one diarrheagenic virulence gene was found in 60/169 (35.5%) isolates. Among the diarrheagenic pathotypes, EAEC was the most prevalent, being present in 29/169 isolates (17.2%). It is presumed that humans are the primary reservoir for EAEC, and the fecal-oral pathway is the primary route through which the strain spreads, causing foodborne and waterborne infections^72–74^. Additionally, a high prevalence of ESBLs in EAEC has been reported globally, demonstrating the mortality of EAEC infections^75,76^. Apart from EAEC, this study also detected multiple diarrheagenic strains, namely ETEC and EPEC in 22/169 (13%) and 9/169 (5.4%) isolates, respectively. ETEC is the major causative agent of diarrhea among children and travelers, and studies have shown that the prevalence of ESBL within these strains may be as high as 35%^77,78^. In contrast to previous studies, this study indicates a higher prevalence of diarrheagenic isolates within FS and DW, which may indicate that current prevention strategies are failing to eliminate such pathogens, leading to continued persistence within the environment. A targeted molecular approach to identify key virulence determinants was used because it provides rapid, specific and reproducible information relevant to pathogen typing^17,28,79,80^. Additionally, 8/169 (4.7%) of the isolates were classified as ExPEC, and the prevalence of these strains is higher than those previously detected, which further strengthens the notion of failing treatment efficiencies within these habitats^16,17^.

The understanding of antibiotic susceptibility profiles of bacteria within environmental reservoirs may assist in selecting antibiotics for intervention and therapeutics. In this study, 166/169 (98.2%) isolates were found to be MDR. Previous studies have shown a similar prevalence of MDR isolates from FS and DW samples^16,17,29,59^. An extended spectrum of resistance was seen in the case of cephalosporins, with a large proportion of isolates being resistant to cefepime, a fourth-generation cephalosporin. Moreover, high resistance levels were also observed in the case of tetracyclines and combination antibiotics. This may be due to the high levels of consumption of these antibiotics within the country^81^. A high level of intermediate resistance was seen in the case of piperacillin-tazobactam, ceftazidime, aztreonam and nalidixic acid. This indicates the future risk of these bacteria possibly acquiring resistance to these antibiotics, rendering them invaluable in the case of therapeutic treatment. Therefore, policies that mandate unregulated consumption of antibiotics must be implemented on a population level to combat the rise in AMR within these environmental reservoirs.

The production of biofilm by *E. coli* on biotic and abiotic surfaces facilitates continued persistence within these environments^82^. The production of biofilm by ESBL-producing *E. coli* induces a favourable environment, for the efficient exchange of antibiotic resistance genes and widespread dissemination of pathogenic genes within the population. Therefore, this study assessed the biofilm formation ability of the ESBL-producing *E. coli* isolates. The isolates were found to form a higher amount of biofilm at 25 °C, and previous studies have shown higher biofilm formation at similar temperatures^83^. The DW isolates were found to form more biofilm at 25 °C in comparison to the FS isolates, with a decrease in biofilm formation at 37 °C. In previous studies, *E. coli* was shown to form a decreased amount of biofilm at similar temperatures in comparison to other waterborne pathogens. The difference in the biofilm formation may be attributable to several aspects such as environmental stress and physicochemical parameters conducive to the procedure^84^. Since the choice of growth medium can influence biofilm formation, our use of LB broth—consistent with previously published protocols—should be considered when comparing biofilm results from this study with those obtained using other media such as Mueller Hinton or Tryptic Soy Broth. The observed variation in biofilm formation at different incubation temperatures has important ecological and public health implications. Biofilm formation at lower temperatures (25 °C) reflects the ability of *E. coli* to persist in environmental matrices. Enhanced biofilm formation under these conditions can protect bacteria from environmental stressors, disinfectants, and nutrient limitation, thereby facilitating long-term survival and increasing the likelihood of environmental transmission. Conversely, biofilm formation at higher temperatures may be relevant to survival within host-associated environments^83,85^.

The dendrogram analysis obtained for the *E. coli* isolates resulted in a total of 16 clusters at a 70% similarity index. The largest cluster ‘O’ contained 50 isolates, and demonstrated the similarities between the isolates despite variations in the phenotypic and genotypic profiles. The findings are similar to studies conducted previously and support the high genetic diversity observed within this study^16,17^. The sequence similarities between the isolates obtained from DW and FS suggest that they may arise from the same clonal lineage, most likely due to contamination extending from a single source. The main inference from this outcome is that ERIC may serve as a method to trace contamination and effectively compare isolates obtained from various related environmental reservoirs. Furthermore, relocation of the FSTPs to a more secluded area further away from the households may serve to prevent excessive contamination seen in this study.

The co-existence of pathogenicity and resistance within ESBL-producing *E. coli* isolates presents an increased risk to the surrounding population. These pathogens may likely escape from these natural reservoirs and cause an endemic in densely populated regions. The present study revealed associations between the presence of pathogenicity markers and resistance, and previous studies have shown ESBL-producing *E. coli* isolates to concurrently harbour MDR pathogenic genotypes^86^. Hierarchical clustering provides a useful approach to group the isolates based on their phenotypic and genotypic traits, as opposed to specific sections of genomic repeats. In this study, several DW and FS isolates were grouped under the same cluster, most likely due to cross spread of the bacteria from FS into DW supplies or vice versa, showing the potential ability of the pathogens to spread between two environmental reservoirs.

The findings of this study are alarming as they highlight the role of DW to serve as a vector for the dissemination of these MDR *E. coli*. The finding of ESBL-producing *E. coli* in the outlets suggests that these bacteria could survive treatment and possibly spread to the surrounding area. Groundwater is the main source of drinking water in the Rohingya camps, which is then stored for consumption at the household level. Indirect cross-contamination between fecal sludge and drinking water sources may be facilitated by the close proximity of sanitation infrastructure, high population density, and environmental fecal contamination, even though these sources do not directly receive treated wastewater from fecal sludge treatment plants. The spread of genetically related isolates between household drinking water and fecal sludge may be facilitated by these circumstances. The contamination of DW with ESBL-producing *E. coli* drives the risk of untreatable infections in the camps, severely affecting children and immunocompromised individuals who already face an increased risk of waterborne diseases. These findings reiterate the urgent need for targeted interventions that prevent cross contamination between FS and DW sources.

There are a few limitations of this study. Firstly, because multiple isolates were recovered from individual samples, and ERIC-PCR was used to assess genetic relatedness, no high-resolution assay was performed to verify that they were not clones. Therefore, fine-scale clonal relationships among isolates from the same sample cannot be fully understood. The lack of whole genome sequencing data limits the inception of our results in broader studies, as sequence data may help to determine the reason behind resistance on a genome wide level. The lack of a conjugation study limits the capacity to test the transferability of genes within the isolates from different environmental reservoirs, leading to a lack of data to understand the dissemination of harmful genes on a population level. Although we used validated PCR assays for virulence gene detection to characterize *E. coli* pathotypes in this study, inclusion of conventional serotyping and whole genome sequencing in future work would provide additional resolution insights into epidemiological linkage and potential clonal dissemination between fecal sludge and drinking water isolates. Finally, the study did not investigate the behavioral patterns of the population causing the spread of these pathogens within the environmental reservoirs. Nevertheless, this study generates useful insights into the presence of endemic causing pathogens within the tested environmental reservoirs, and future studies addressing the limitations are necessary.

In conclusion, this study has identified a high prevalence of ESBL-producing *E. coli* within the fecal sludge samples and proximal drinking water within Rohingya camps. The isolates were found to harbor high incidences of resistance and pathogenic genes, highlighting the potential disease causing ability of the bacteria with limited treatment prospects. The dissemination of pathogenic *E. coli* aided by these environmental reservoirs leads to continued disease prevalence within these housing communities. To combat this, targeted interventions that decrease prevalence such as routine cleaning of drinking water storage tanks and water disinfection processes such as chlorination or ozone treatment prior to consumption may help aid in decreasing contamination. For fecal sludge, treatment processes such as lime stabilization ponds and incineration of treated fecal sludge that essentially eliminate any organic matter must be prioritized over other technologies. On a broader scale, the non essential use of antibiotics must be limited to prevent pathogens from acquiring drug resistance. This study is an essential call to change these policies and advocate for an integrated, genomics-based approach to mitigating AMR in resource constrained areas.

## Supplementary Information

Below is the link to the electronic supplementary material.

Supplementary Material 1

Supplementary Material 2

Supplementary Material 3

Supplementary Material 4

Supplementary Material 5

## Data Availability

Data will be made available on request from the corresponding author, Zahid Hayat Mahmud (zhmahmud@icddrb.org).
