# Supplementary material for: Fecal sludge and adjacent drinking water as reservoirs of multidrug resistant ESBL-producing pathogenic Escherichia coli in Rohingya Camps, Bangladesh
Source: Sci Rep. 2026 Jun 22;16:28503. doi: 10.1038/s41598-026-58907-y (PMC13572393; doi:10.1038/s41598-026-58907-y)
Supplement: Supplementary file 1 [file 41598_2026_58907_MOESM1_ESM.docx]

**Figure S1. PCR for ESBL genes (*bla_CTX-M_*, *bla_TEM_*, *bla_SHV_* and *bla_OXA_*)**

**TEM**

**SHV**

**CTX-M**

**OXA**

L 49 50 55 56 57 59 60 61 62 63 66 67 - 68 76 51 69 52 53

L (-) (+) (+) (+) 23 24 25 26 38 39 40 41 42 43 44 45 46 47 48

[L= Ladder; (-) = Negative control; (+) = Positive control; The numbers indicate isolate IDs]

**Figure S2. PCR for diarrhoeagenic pathotypes (*aat, aaiC, eae, bfpA, eltB*, *estA, ipaH, iaa*)**

***iaa***

***aaiC***

***ipaH***

***eltB***

***estA***

***bfpA***

***eae***

15 16 17 18 19 20 21 22 L

L (+)(+) (+) (+) (-) 1	2	3	4	5	6	7	8	9	10 11 12 13 14

[L= Ladder; (-) = Negative control; (+) = Positive control; The numbers indicate isolate IDs]

**Figure S3. PCR for ExPEC**

L 38	39	40	41	42	43	44	45	46	47	L	(-)	19	20	21	22	23	24	25

[L= Ladder; (-) = Negative control; The numbers indicate isolate IDs]

**Figure S4. PCR for *bla_CTX-M-1_* gene**

(-)	L	(+) 72 73	74	75	76	77	78 79	80	81 82	83	84	85	86	91

[L= Ladder; (-) = Negative control; (+) = Positive control; The numbers indicate isolate IDs]

**Figure S5. ERIC PCR**

(-)	L	29	30	31	32	33	34	35	36	37	38	L

[L= Ladder; (-) = Negative control; The numbers indicate isolate IDs]
